# Biocomposites Based on Plasticized Wheat Flours: Effect of Bran Content on Thermomechanical Behavior

**DOI:** 10.3390/polym12102248

**Published:** 2020-09-29

**Authors:** Franco Dominici, Francesca Luzi, Paolo Benincasa, Luigi Torre, Debora Puglia

**Affiliations:** 1 Civil and Environmental Engineering Department, University of Perugia, Strada di Pentima 4, 05100 Terni, Italy; franco.dominici@unipg.it (F.D.); francesca.luzi@unipg.it (F.L.); luigi.torre@unipg.it (L.T.); 2 Department of Agricultural, Food and Environmental Sciences, University of Perugia, Borgo XX Giugno 74, 06121 Perugia, Italy; paolo.benincasa@unipg.it

**Keywords:** bran content, plasticized wheat flour, citric acid, biobased blends

## Abstract

In the present work, the effect of different bran content on the overall thermomechanical behavior of plasticized wheat flours (thermoplastic wheat flour; TPWF) was investigated. Refined flour (F0) with negligible bran fiber content, F1 flour (whole grain flour, 20% wt. bran), F3 (50% wt. bran) and F2 (F1:F3, 50:50) film samples were realized by extrusion process. The effect of TPWF blending with two different biopolymers (polycaprolactone and poly butyrate adipate terephthalate), combined with the presence of citric acid as compatibilizer was also considered. Results from FESEM analysis and tensile characterization demonstrated that PCL was able to reach improved compatibility with the plasticized flour fraction at intermediate bran content (F2 based formulation) when 25% wt. of biopolymeric phase was added. Additionally, it was proved that improvements can be achieved in both thermal and mechanical performance when higher shear rate (120 rpm) and low temperature profiles (T_set_2 = 130–135–140 °C) are selected. Disintegrability of the TPWF basic formulations in compositing conditions within 21 days was also confirmed; at the same time, an absence of any phytotoxic event of compost itself was registered. The obtained results confirmed the suitability of these materials, realized by adding different bran contents, to mechanically compete with bioplastics obtained by using purified starches.

## 1. Introduction

The increasing cost of petrol-based plastics and the public concern about their contribution to environmental pollution have raised the interest towards biobased and biodegradable materials, which help to dispose of by-products from agricultural production and food industries. In the case of bioplastics, purified starch from many agricultural sources (e.g., cereals, tubers, etc.) is often used as a basic ingredient. However, there is literature on the use of wheat flours to obtain bioplastics as an energetically and economically cheap alternative to purified starch [1,2]. Previous research from our group demonstrated that the tensile properties of thermoplastic films depended on wheat grain hardness and baking properties of refined flours [3,4]. However, the use of wholegrain flours has received limited attention, even if this approach could be of relevance for the reinforcement effect, due to bran, on the overall performance of plasticized starches [5]. Bran represents the outer portion of the grain, including the pericarp and seed teguments, containing a relevant amount of lignin and cellulose, the so-called fiber. It accounts for around 15–25% wt. of the total grain weight and generally comes out as a by-product of grain milling. It is normally used as animal feed [6]; however, the progressive decrease of the whole national livestock that occurred in the last decade has led to an increase of bran stocks to get rid of, with possible valorization as biological chemicals and energy source [7]. The effect of bran particle size on functionality of the gluten network was already explored in wholegrain flour and its baking properties [8]: It was found that a deleterious effect on time of dough development, gluten strength, starch gelatinization and the retrogradation was intensified by the presence of all constituents of the grain in the wheat mass formulation when compared to refined flour. In particular, it was evidenced that the quality of the protein and the differences between the particle sizes with respect to the stability and development time are broadly correlated with the quality of the gluten network. Additionally, it was proven that the presence of fibers limited the availability of water to the starch in the wholegrain samples, and that this effect was especially strong for flour with finer particle size, which also had the highest rate of absorption.

In another paper, Liu et al. [9] studied the adverse effects of wheat bran on gluten network formation, which may lead to the reduction in gluten viscoelasticity and quality deterioration of fiber enriched flour products. With the properties of starch, such as degree of gelatinization, gel stability, and retrogradation, being strongly influenced by the availability of water in the formed mass system [10], it is considered extremely important to investigate the role of bran content and its grinding level even on the thermomechanical behavior of plasticized flours.

Since it is expected that the bran level could, in general, increase the strength of the matrix to the expense of the deformability of the plasticized flour [11] by creating macroscopic defects in the material, the possibility of using biobased polymers in combination with thermoplastic flour to recover the plasticity has been also considered. The literature reports results on the effect of fiber reinforcement on mechanical behavior of thermoplastic starch blended with different polyesters [12], but no examples are available on how the presence of bran additive could tune the mechanical behavior; according to this, we here attempted to verify, for the first time, how different contents of grinded bran could affect the deformability of the flour; furthermore, blending with low melting polymeric fractions (polycaprolactone and polybutylene adipate terephthalate) was considered to increase the limited deformability of the polymeric matrix.

## 2. Materials and Methods

### 2.1. Materials

Soft wheat produced in the Umbria region (Italy) was chosen as the reference wheat variety to obtain, by grinding and selective extraction, flours with different bran contents. The milling products were kindly supplied by Molini Spigadoro (Bastia Umbra, Italy). The chemicals, glycerol, magnesium stearate, D-sorbitol, water, polyvinyl alcohol (≥99% hydrolyzed) (PVA) and citric acid (CA) were supplied by Sigma Aldrich. Polybutylene adipate terephthalate (PBAT) Ecoflex F Blend C1200 was supplied by BASF. Polycaprolactone (PCL) Capa 6500 was kindly provided by Perstorp.

### 2.2. Preparation of Milled Products, Their Plasticization and Blending

Four wheat flour milling products, namely F0, F1, F2, F3, were considered. The detailed compositions are reported in Table 1.

F0 was a refined flour with negligible percent bran content; F1 was a wholegrain flour as it would come out from milling the whole grain (including the pericarp and seed coats); F3, with a bran content of 50% (wt.), represented the outcome generally obtained as the grinding tail of milling; F2 was obtained by mixing F1 and F3 in equal parts (50:50 wt.).

Plasticization of the samples was carried out with an Xplore Microcompounder 5 & 15 cc extruder (DSM, Sittard, The Netherlands) by considering suitable contents of glycerol, water and other process facilitating additives, as reported in our previous work [3]: Flour (68%, *w*/*w*), glycerol (23%, *w*/*w*), magnesium stearate (1.8%, *w*/*w*), sorbitol (5.2%, *w*/*w*), PVA in aqueous solution PVA/water 1:20 (2%, *w*/*w*). The initial process parameters were set as reported in our previous paper on refined flours: Temperature profile (T_set_2) in the three heating zones of the extruder at 130–135–140 °C and mixing at 30 rounds per minute (rpm) for 6 min [4].

The doses of the reagents for the plasticization were adapted by calculating the plasticizable fraction of material (PF) (starch, proteins and other components), excluding the fiber and the other not plasticizable constituents (UF). Indeed, fiber content, which represents the fibrous portion of flour, does not participate to the plasticization process, while the bran content, which has plasticizable fraction, should be taken into account. The fiber, not participating directly in the plasticization reaction, was considered in the formulation only for the evaluation of absorbed water, estimated to be at 15% by weight of fiber [13]. In order to give an explanation of the adopted methodology for plasticization, a detailed recipe for the F1 sample is given as an example. F1 flour, as typical wholegrain flour, consists of 80% flour and of 20% bran. The plasticizable fraction (PF) of F1 is 86% wt. (given by 0.8 × 1.0 + 0.2 × 0.3, where 1.0 is the PF of the flour and 0.3 is the PF of the bran). In a similar way, PF values have been calculated for all the samples and summarized in Table 1.

In the case of blends based on TPWF, two types of biodegradable polymers, PBAT and PCL, were initially considered at a weight amount of 20% wt. In the case of polycaprolactone, the research was extended to blends containing 25, 30 and 40% wt. of the polymeric component. Further attempts to optimize the formulations were made by changing the quantities and types of plasticizers. The amount of glycerol was reduced from 23 to 17% wt. and, at the same time, an additional water fraction of 17% wt. was added to provide hydroxyl groups functional to plasticization, less available due to glycerol reduction. Moreover, the plasticizing and compatibilizing effect of citric acid added at 0.8% wt. was also evaluated. Then, an optimization of the parameters was tried by varying the temperature and mixing speed. The effects of increasing the temperature profile were tested by setting T_set_3 to 135–140–145 °C. An attempt was also made increasing the rotation speed of the screws from 30 to 120 rpm. All the samples were used to produce specimens of film with a thickness of about 300 µm with the aid of a Film Device Machine (DSM, Sittard, The Netherlands) coupled to the extruder. The list of samples is summarized in Table 2.

### 2.3. Characterization of Flours and TPWF-Based Composites

#### 2.3.1. Alveographic Properties

F0 flour was used for the measurement of alveographic parameters as it is a good approximation to the plasticizable fraction of the processed samples. The tests were carried out by using a Chopin alveograph (Alveolink NG, Villeneuve-la-Garenne, France) in constant hydration (HC) mode, following the recommendations of the ISO 27,971 standard. Average values of the main alveographic parameters, Tenacity (P), Extensibility (L), Baking strength (W), and Configuration ratio (P/L), were determined with five replicates.

#### 2.3.2. Thermogravimetric Analysis

Thermal degradation of the milling products F0, F1 and F3, having different content of bran, was evaluated carrying out thermal dynamic tests, from 30 °C to 600 °C at 10 °C min^−1^ by thermogravimetric analysis (TGA, Seiko Exstar 6300, Tokyo, Japan). About 5 mg of each sample was used, and dynamic tests were performed under nitrogen flow (200 mL min^−1^). Mass loss (TG) and derivative mass loss (DTG) curves for each tested material were evaluated.

#### 2.3.3. Tensile Tests

A universal electronic dynamometer LR30K Plus (LLOYD Instruments, Bognor Regis, UK) was used to carry out a mechanical characterization of the materials. Tensile tests were performed by setting a crosshead speed of 5 mm min^−1^ on 20 × 150 mm rectangular specimens about 300 mm thick, in accordance with ISO 527 standards. Ultimate tensile strength (σ) and strain at break (ε_b_) were calculated from the resulting stress–strain curves with the support of a software specific to the test machine: NEXYGEN Plus Materials Testing. The measurements were done, after conditioning the samples at room temperature for 24 h at 50% relative humidity (RH), testing at least five specimens for each formulation.

#### 2.3.4. Morphological Evaluation

A first visual analysis was performed on TPWF/bran-based film samples. Moreover, a morphological characterization of composites was carried out using a field emission scanning electron microscope (FESEM) Supra 25 by Zeiss (Oberkochen, Germany). Micrographs of fractured surfaces obtained by cry fracturing the samples in liquid nitrogen were taken with an accelerating voltage of 5 kV at different magnifications. Previously, the samples were gold sputtered to provide electric conductivity.

#### 2.3.5. Disintegration in Compost

The compost mineralization of the films was evaluated on the basis of the ISO 20,200 standard. A certain amount of compost inoculum, supplied by Gesenu Spa, was mixed together with synthetic organic waste, prepared with an appropriate amount of sawdust, rabbit feed, starch, sugar, oil and urea to thus constitute the soil for composting. The soil moisture content was maintained at values of 50% RH by adding water and mixing at regular intervals of time, as indicated by the legislation, while aerobic and thermal conditions were guaranteed during the test. Based on ISO 20200, a sample can be considered disintegrated when it reaches 90% mass disintegration in at least 90 days in contact with the composting soil in the ripening phase. The disintegration percentage after a time *t* in compost is calculated as reported in Equation (1):

(1) $$D_{t}=\frac{m_{i}-m_{r}}{m_{i}}\times 100$$

where *m_i_* is the initial mass of the sample and *m_r_* is the mass of the extracted sample, after drying, at a given time *t*.

#### 2.3.6. Evaluation of Phytotoxicity

The phytotoxicity of the compost obtained from the disintegration test of the films was assessed at 40 days from the start of composting and, following the obtained results, the evaluation was repeated at 60 days. A germination test was carried out on cress seeds (*Lepidum sativum* L.), a test plant normally used for this purpose, as required by the IPLA, DIVAPRA, ARPA methods “Compost Analysis Methods”, 1998. This method involves the evaluation of the effect of an aqueous extract of compost, picked up from disintegration tests, on seed germination. It was decided to evaluate three composts, those obtained with plastic films derived from flours F0, F1 and F3, assuming that F2 would give an intermediate result between F1 and F3. For each compost, the following standard procedure was used. Each sample to be tested (200 g) was brought to a humidity of 85% and left for two hours in contact with the added water. It was then centrifuged at 6000 rpm for 15 min and the supernatant was filtered under pressure at 3.5 atm with a sterilizing membrane. The aqueous extract was diluted up to concentrations of 50% and 75%. Five aliquots, each of 1 mL, of each of the two dilutions of the obtained samples (plus the same number of controls with water) were placed in 9 cm diameter Petri dishes containing bibulous paper. 10 seeds of *Lepidium Sativum* were added to each capsule, soaked for one hour in distilled water. The capsules were placed to incubate at 27 °C for 24 h. After this period, the germinated seeds were counted, and the root length of the buds was measured. The germination index (*I_g_*) was calculated as indicated in Equation (2):

(2) $$I_{g}\left(\%\right)=\frac{\left(G_{c}\;\times \;L_{c}\right)}{\left(G_{t}\;\times \;L_{t}\right)}\times 100$$

where:

*G_c_* = average number of germinated seeds in the sample
*G_t_* = average number of seeds germinated in the control
*L_c_* = average root length in the sample
*L_t_* = average root length in the control

The values of the germination indices for 50% and 75% dilutions after 40 and 60 days of maturation of the compost extracted from the disintegration soils of the samples TPF0, TPF1 and TPF3 were analyzed.

#### 2.3.7. Statistical Analysis

Data were analysed by analysis of variance (ANOVA), using the Statgraphics Plus 5.1. Program (Manugistics Corp. Rockville, MD, USA). To differentiate samples, Fisher’s least significant difference (LSD) was used at the 95% confidence level.

## 3. Results and Discussion

### Wheat Flour Characterization

The refined flour F0, having a moisture content of 14.5% wt. and a protein amount of 11.8%, was tested with the Chopin’s alveograph at constant hydration (HC), showing the following alveographic parameters: Tenacity (P = 64 mm H_2_O), extensibility (L = 99 mm), baking strength (W = 182 × 10^−4^ J), configuration ratio (P/L = 0.65), elasticity index (I_e_ = 47.4%). These alveographic parameters are characteristic of a standard flour with moderate strength and standard quality for basic baking uses.

**Figure 1:**
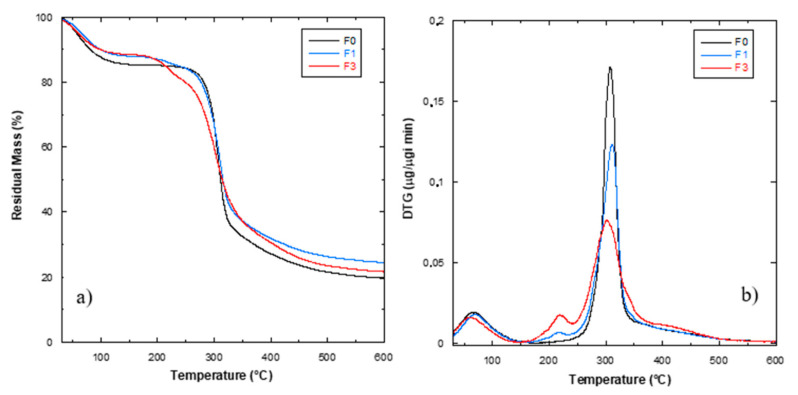
Mass loss (TG) (**a**) and derivative mass loss (DTG) (**b**) curves of F0, F1 and F3 samples.

The results of the thermogravimetric analysis (Figure 1) show that, with the exception of weight loss due to water evaporation at around 80 °C, there were no significant weight losses due to thermal degradation within the temperature range for plasticization up to 150–160 °C. As reported in [14], the shape of this low temperature peak can be varied due to bran addition: It was shown that flour rich mixtures exhibit distinct features with an initial peak attributed to starch and a secondary shoulder attributed to gluten. In general, they observed that a gradual shift occurred in the gluten shoulder, in conjunction with the addition of bran to the mixture. In our case, we observed that in bran rich mixtures, i.e., F3 formulation, the peak was not symmetric and a shift to a lower temperature range was observed due to a modified moisture release from the flour during heating.

The thermogravimetric analysis evidenced also the typical degradative pattern for cereal flours. The three wheat flours showed similar TG curves, with small differences in terms of residual weight at the end of the test, in line with the typical mass loss values (17% *w*/*w* for wheat flour) and the additional residue due to the presence of the bran component (Figure 1a): After water evaporation, the second main step centered at 300 °C corresponds to the decomposition of starch, while the third step (T > 400  °C) corresponds to the formation of inert carbonaceous residues [1] (Figure 1b). In the case of increasing bran amount, the F1 and F3 samples showed the presence of a further peak, in the range 200–250 °C, identified as the starting point for decomposition of lignocellulosic components (mainly cellulose and hemicellulose) in the bran fraction [15].

**Figure 2:**
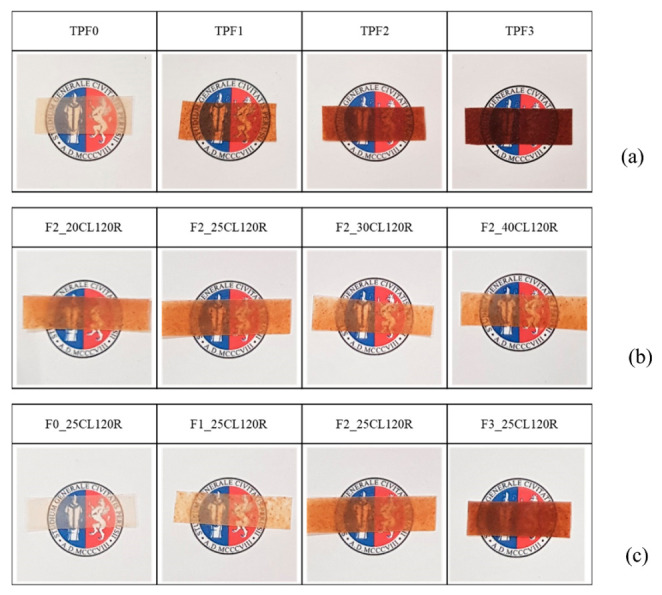
Visual observation of (**a**) thermoplastic wheat flour (TPWF) based films made with flours with increasing bran content (from F0 to F3), (**b**) F2 based films blended with 20, 25, 30 and 40% wt. of PC and (**c**) films based on plasticized F0, F1, F2 and F3 blended with 25% wt. of polycaprolactone (PCL).

Having established that the flours with variable bran content could be processed and plasticized in the selected temperature range without losing thermal stability, films based on F0, F1, F2 and F3 flours were realized by extrusion, as detailed in Section 2.2. The visual analysis of the films in the top row (Figure 2a), produced without the addition of biopolymers, showed a progressive browning as the fraction of bran in the flour increased. However, films with low fiber content showed good transparency, satisfying a fundamental characteristic for some packaging applications. The yellowing/browning of the TPWF films, which it is normally caused by the non-enzymatic reactions that occur during plasticization, is emphasized by the presence of the bran, which adds opacity and darkening [16,17,18].

In Figure 2b, the effect of the addition of PCL in the formulation based on F2 flour produces an improvement in transparency, which was enhanced as the proportion of biopolymer in the blend increased. In the last line of the picture (Figure 2c), images of the films obtained after the optimization of compositions (25% wt. of PCL fraction) and processing parameters (120 rpm) are included. The film based on refined flour F0 shows good transparency, which remained acceptable even in the F1-based film, despite the yellowing due to the presence of bran fibers. The level of transparency of F2_25CL120R is also acceptable, although the darkening caused by the abundant bran fraction produced a color change on browning tones and a sensible reduction in transparency [19]. In the case of F3-based film, the transparency was compromised to an extent that makes the film unsuitable for applications requiring visibility of the underlying objects, while its use for opaque packaging or other applications, such as mulching or shading sheets, where opacity is functional, can be envisaged for the F3_25CL120R composition.

**Figure 3:**
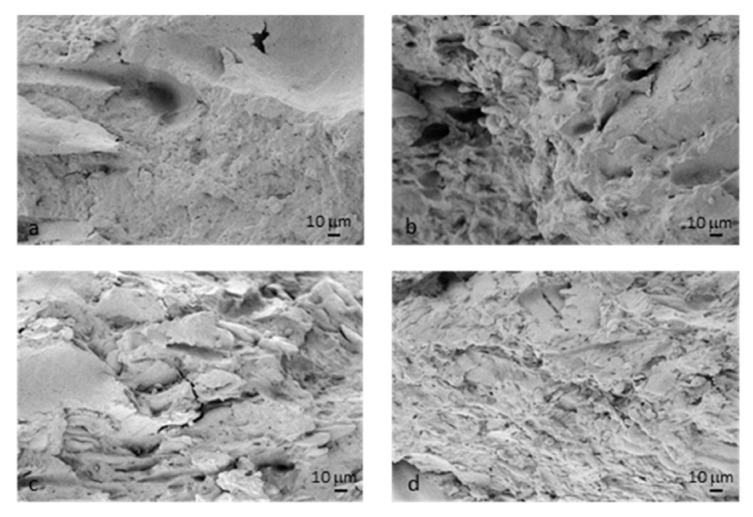
FESEM images of fractured cross-sections of (**a**) TPF0, (**b**) TPF1, (**c**) TPF2 and (**d**) TPF3 films, acquired at 1000× magnification.

The morphologies of the fractured surfaces for the TPWF films were observed by FESEM (Figure 3) and differences were found for the four milling products with different bran contents. In detail, the F0 flour (Figure 3a) appeared well plasticized with a uniform surface, no separate starch granules were noted and the absence of bran particles was evident. Plasticization of the F1 flour (Figure 3b) was also well achieved, since a smooth and homogeneous plastic phase was found in the analyzed surface. Bran fibers with a particle/lamellar appearance were uniformly distributed and well bonded to the plasticized starch, suggesting the realization of a composite material with good characteristics. Similarly to the previous ones, the plasticization of the F2 flour was also performed with good results.

In this case (Figure 3c), we noted the prevalent presence of lamellar particles of bran that resulted oriented, as alternating layers with the plastic phase, due to the orienting effect of the production process. F3, with the highest fiber fraction among the selected flours, highlighted the prevalent presence of bran particles (Figure 3d), with the plasticized starch having reduced adherence to the bran particles. Fibrous agglomerates and not well plasticized starch particles were noted: Due to the hindering effect of the large amount of bran fiber, wheat flour granules were less capable of forming hydrogen bonds with plasticizers through their hydroxyl groups, leaving some domains unreacted with unplasticized starch particles [3,20]. In general, while observed morphologies in TPF1 and TPF2 can confirm the ductility of the films, to the expense of low strength (that actually increased in TPF2 due to increased filler content), TPF3 appears saturated with the reinforcement phase and a matrix phase close to the wettability limit of the fibers. In this case, behavior that maximizes strength and stiffness but limiting the elastic-plastic characteristics can be expected.

The observations made by analyzing the sample morphologies were confirmed by checking the results of tensile characterization made on the same series of materials In Table 3, the results of the tensile tests carried out on plasticized flour samples and their bioblends are included.

**Figure 4:**
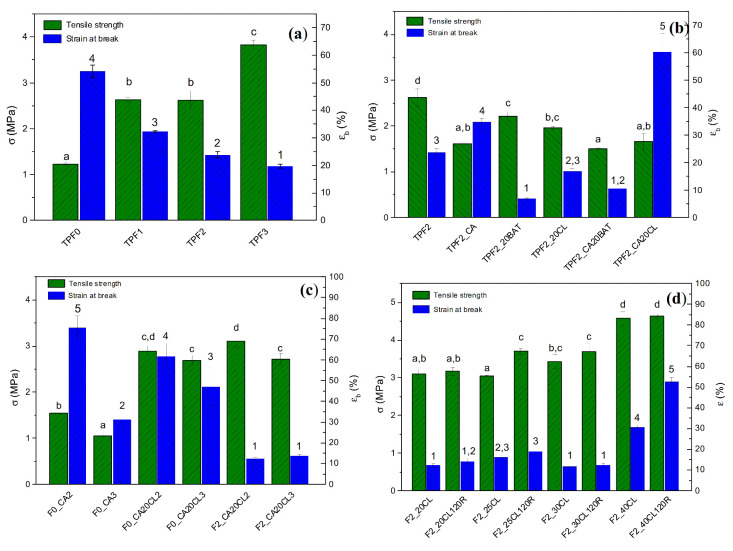
Results of tensile strength and strain at break for (**a**) TPWF/bran film samples, (**b**) TPWF/bran and biopolymeric blends, (**c**) samples processed with two different plasticization temperature profiles (T2, T3), (**d**) samples processed with two different mixing rates (30 rpm, 120 rpm). (a–d, stress values) (1–5, strain values) Different superscripts within the same column group (stress or strain values) indicate significant differences among formulations (*p* < 0.05).

The refined F0 flour, following the plasticization process, shows mechanical properties in line with other flours, with comparable alveographic characteristics, tested in previous works [3]. As evidenced in Figure 4a, good elongation values (54%) correspond to a moderate tensile strength (1.23 MPa), which is the main drawback of TPWFs. The selection of flours containing different bran fractions offers the advantage of having a fibrous filler, which can be effective as a reinforcement phase and, at the same time, has a plasticizable fraction, able to guarantee good compatibility and bonding at the interface with the starch matrix upon plasticization. The bran plays the role of reinforcement by preventing creep and deformation of the thermoplastic phase. As the bran fraction and consequently the fiber content increased, the samples showed decreasing strain values. TPF1 showed an ε_b_ of 32.2%, which decreased to 23.8% with TPF2, further dropping to 19.6% in the case of the TPF3 sample. On the other hand, when the percentage of bran increased, the tensile strength increased as well, reaching an σ value more than tripled in the case of the F3-based sample (3.83 MPa) when compared to refined F0 flour.

The tensile strength of TPF1 was more than doubled (2.63 MPa) compared to the sample without fiber TPF0; TPF2 shows the same tensile strength value (2.62 MPa) as TPF1, albeit with a higher bran content. This result suggested the possibility of an improvement of the mechanical properties for reference TPF2, which could be achieved by improving the dispersibility of the bran fiber in the plasticized matrix. To pursue this goal, the characteristics of the matrix must be enhanced by improving the compatibility with the reinforcement phase. Comparing the values obtained for all formulations, it can be commented that values for the maximum tensile strength and elongation at break changed significantly (*p* < 0.05).

In order to further improve the characteristics of the F2 based TPWF, two actions were considered: The improvement of the intrinsic characteristics of the composite by using plasticizers/compatibilizers and the addition of another matrix in blend that could enhance the characteristics of the entire composite system. The selection of the matrices was carried out taking into account some preliminary criteria, such as physical–chemical compatibility, conservation of the biodegradability of materials and process compatibility, possible plasticization and blending in one step to minimize energy waste and environmental impact, according to an eco-sustainable development perspective. Polybutylene adipate terephthalate (PBAT) and polycaprolactone (PCL) were selected for this specific purpose and initially used at a nominal percentage of 20% wt. Citric acid (CA) was indeed considered as a suitable compatibilizer for TPWF: CA, other than having a plasticizing effect, can be also effective in the compatibilization between plasticized starch, bran fiber and biopolymers [21,22]. A nominal percentage of 0.8% wt. was chosen, on the basis on the results of previous literature works [23]. The effects on the composites were firstly evaluated by adding individually CA, PBAT and PCL, and then the formulations with the concurrent use of biopolymer and compatibilizer were studied.

In Figure 4b, it is demonstrated that the citric acid, added alone to the TPF2 formulation, has a plasticizing effect, increasing the deformation to 34.9% but reducing the strength to 1.61 MPa. The TPF2_20BAT blend showed an increase in tensile strength, beside a reduction in deformability, which highlighted the poor compatibility between TPWF and PBAT. A decrease of both values of strength and deformation at break was also found in the TPF2_20CL formulation, with only PCL in blend. The use of citric acid was found to have no positive effects when added in the presence of PBAT, while its role was effective when combined with PCL. TPF2_20CL showed an increase in strain at break up to 60.4% compared to the value of 16.8% for the same sample without CA. The addition of citric acid induced reactions able to favor the adhesion between PCL and TPS (trans-esterification), improving the wettability (hydrolysis) and inhibiting the formation of cross-linking (sulfhydryl (SH)-SS exchange) during flour plasticization [21,22,24,25,26].

Considering that glycerol has a much higher plasticizing effect, due to the presence of three hydroxyl groups, compared to water, and even a “lubricating” effect that lowers the stiffness of the plasticized system, it was planned to replace 6% wt. of glycerol with 17% wt. of water [27]. Furthermore, an attempt was made to improve the mechanical performance of the produced films by varying the plasticization temperature from T2 to T3 [4,28]. The increase of the temperature had the purpose of improving the tensile stress resistance of the materials, by intensifying the formation of bonds and crosslinking, typical of the plasticizing process, conferring strength and rigidity to the TPWF. To better understand the effect of these variations on mechanical properties of TPWF-based samples, both the samples of refined flour F0 and those of F2 flour were tested. The three pairs of samples processed at T2 and T3 (Figure 4c) showed that the increase in temperature, in the presence of water and CA, generally causes a worsening of the mechanical properties, by lowering the stress and strain values. At higher temperatures, the hydrolytic phenomena induced by CA prevailed over the effects of transesterification and cross-linking, supported by the kinetics of the plasticization reaction of the flours at T3. It should be noted that the new dosage with the partial replacement of glycerol with water produced a notable increase in strength (+74%), that moved from 1.66 MPa of TPF2_CA20CL to 2.89 MPa of F2_CA20CL as expected.

**Figure 5:**
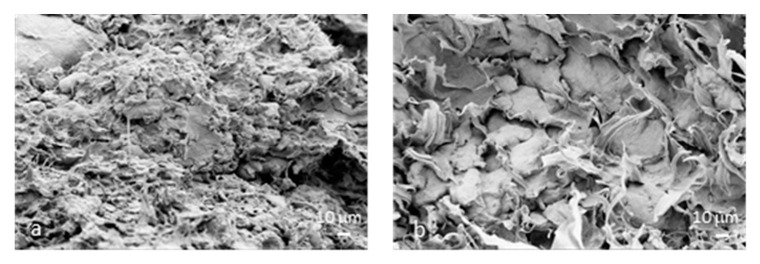
FESEM morphologies of F2_25CL30R (**a**) and F2_25CL120R (**b**) samples.

A further attempt to optimize the formulations was made by increasing the fraction of PCL in the blend to evaluate the ideal TPWF/PCL ratio (Figure 4d). Furthermore, the effect of shear stresses during plasticization was evaluated by processing samples at 30 and 120 rpm. It is known that the shear stresses applied during the plasticization phase can produce effects on the destruction of starch granules and, consequently, on the mechanical characteristics of the materials. In order to take into account the effect of the rheological characteristics of the system, the tests were repeated for materials with different PCL fractions [29,30]. A higher speed of rotation of the screws during plasticization in the extruder produced a general improvement of the mechanical properties, both in terms of strength and, albeit to a lesser extent, of strain. In particular, the increase of shear stresses raised the strength (+23%) from 3.0 to 3.7 MPa in the sample F2_25CL120R. In Figure 5, the SEM micrographs of the samples processed at different screw speeds show a completely different morphology; higher shear stresses, produced with 120 rpm of screw speed, improved plasticization (Figure 5b); resulting images showed smoother and more uniform fracture surfaces, free of granules and fibrous conglomerates, with uniform distribution of the separate phases of PCL and TPWF.

**Figure 6:**
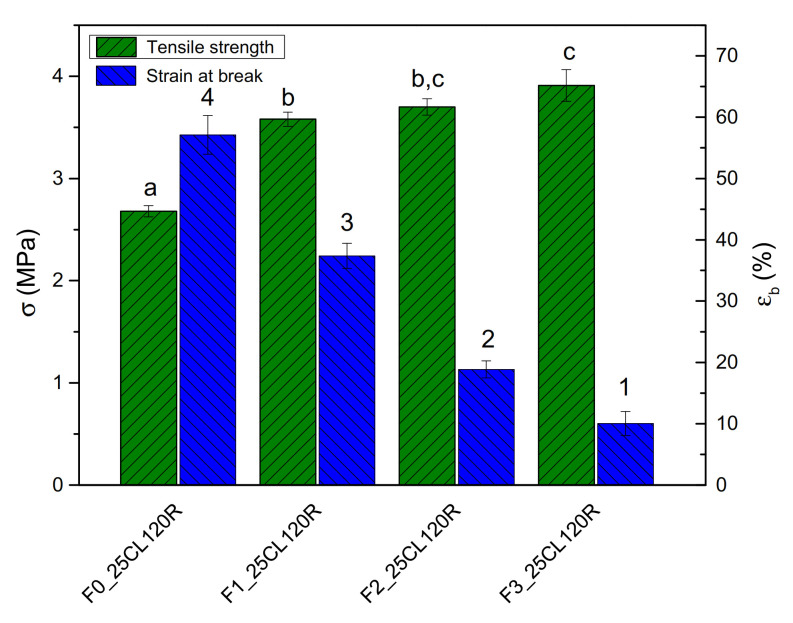
Results of tensile strength and strain at break for TPWF/PCL blends based on F0, F1 and F3 flours, containing 25% wt. of PCL and processed at 120 rpm. (a–c, stress values) (1–4, strain values) Different superscripts within the same column group (stress or strain values) indicate significant differences among formulations (*p* < 0.05).

Finally, samples of all flours were produced using the optimized formulation and process parameters. In Figure 6, the progressive improvement in tensile strength is closely related to the increase in the fraction of bran fiber. The lowest σ value (2.68 MPa) was obtained for F0_25CL120R, which was free of fiber, and rose to 3.91 MPa with F3_25CL120R. The intermediate fiber contents also corresponded to intermediate strength values for F1_25CL120R and F2_25CL120R, equal to 3.58 and 3.70 MPa, respectively. On the other hand, the increase in the bran fraction produced a progressive decrease in the strain at break, which reached 57.1% with F0 flour and dropped to 10.0% for the F3 flour-based material. Samples on F1 and F2, flour-based, showed intermediate ε_b_ values of 37.4% and 18.9%, respectively. A clear effect of the bran fiber fraction on the mechanical properties of the TPWF-based composites was noted: The addition of fiber produced a 45% increase in strength but caused a drop to 18% of maximum elongation reached without fiber. This result suggests that the composite F3_25CL120R has reached the fiber saturation (35% wt.) and a further increase of the reinforcement phase would lead to brittle behavior of the material. Materials made with quantities of fibers between the tested extremes show intermediate values of σ and ε_b_ indicating the possibility of designing the mechanical properties of the material to be produced according to the formulation.

**Figure 7:**
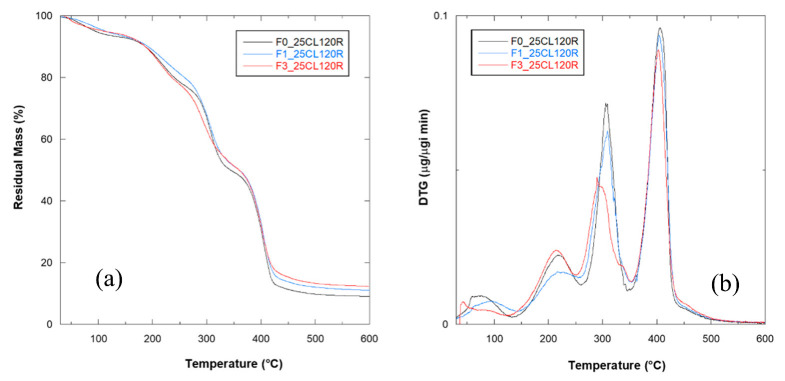
TG (**a**) and DTG (**b**) curves for TPWF/PCL blends based on F0, F1 and F3 flours, containing 25% wt. of PCL and processed at 120 rpm.

The thermal stability of the optimized formulations was determined by thermogravimetric analysis. Figure 7 presents the thermal degradation profile (TG/DTG curves) of the TPWF/PCL blends based on F0, F1 and F3 flours, containing 25% wt. of PCL and processed at 120 rpm. Thermal degradation of blends presented four mass loss stages (Figure 7). Up to approximately 130 °C, there is a mass loss due to the presence of water, while following weight loss, observed between 130 and 230 °C, can be related to the evaporation of glycerol and other volatile compounds present in TPWF [31]. Then, the starch chains began to degrade at about 230 °C [32]; after that, the fourth stage of thermal degradation of the blends occurred from 350 to 430 °C, due to the degradation of PCL chains. The main differences in these profiles was found for the signal of the plasticized TPWF; while the maximum degradation rate of the polymeric PCL phase was almost constant in intensity for all the three blends, the second main weight loss accounted for the reduced amount of plasticized fraction. It essentially followed the trend that TPWF with more bran content (F3 based blend) showed reduced degradation rates. The increased amount of bran was also responsible for increased value of remaining mass at the end of the test, as observed in Figure 7b, due to the charred fraction of fiber.

**Figure 8:**
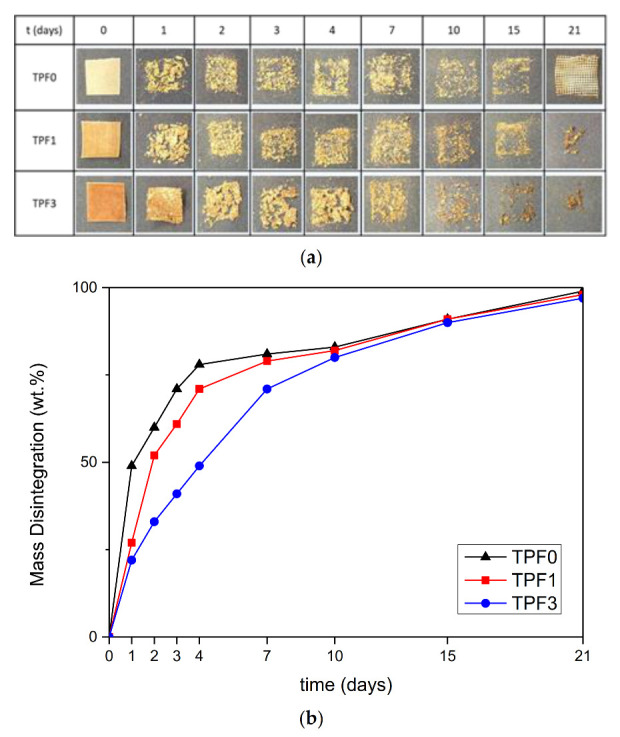
Visual images of the TPWF samples based on F0, F1 and F3 flours (**a**) and evolution of disintegration degree (**b**) at different compositing times.

Figure 8a shows the visual images of the samples during the progress of the disintegration process under composting conditions, while Figure 8b shows the trend of the mass disintegration rate in compost for the three tested formulations. All materials reach 90% disintegration after 15 days under composting conditions. The TPF3 film showed different disintegration kinetics, presenting lower disintegration values, in comparison with TPF0 and TPF1 films, between the 2nd and 4th day under composting conditions. This behavior can be justified considering that higher fiber content was present in F3 film, which slowed down the decomposition process of the plasticized fraction. Starting from the 10th day of the test, both degradation kinetics and final disintegration degree of the three systems were aligned and samples completely disintegrated within 21 days, confirming the compostability, at lab conditions, of the studied materials.

The results obtained 40 days after the start of composting (Table 4) indicate an effect of both the type of compost and the concentration of the extract. All the composts tested were found to have a depressing effect on the germination and growth of watercress sprouts, as the germination index, I_g_ (%), which was always less than 70%, considered the minimum acceptable value. For all compost, a lower germination index corresponded to a higher concentration of the extract. Among the compost, the sample derived from refined flour (F0) is the one that gave the greatest phytotoxic effect, while the compost obtained from both plasticized flours containing bran contents (TPF1 and TPF3) were found to have a less depressing effect on germination performance, but still more toxic than desired. According to this, it was assumed that the revealed phytotoxicity was due to the incomplete maturation of the compost. For this reason, the germination test was repeated with compost extract taken 60 days after composting. In these conditions, it can be seen that, at 60 days, all compost allowed an acceptable germination index (i.e., >70%). In particular, no compost inhibited germination, which was always close to 100% even at the highest extract concentration, while root growth on compost extract obtained from refined flour (F0) was slightly reduced, but always within acceptable limits. On the other hand, there was a kind of hormetic or stimulating effect of the compost extract obtained from F1 flour when used at the lowest concentration (50% dilution); in this case the Ig (%) was 108%. This result is not surprising because it is known in the literature that various substances, both synthetic and natural (e.g., NaCl and other salts, herbicides and allelopathic substances), can have a depressive effect at high concentrations or a stimulating effect at low concentrations.

## 4. Conclusions

The objective of this work was the study of thermomechanical behavior of eco-sustainable and biodegradable materials obtained by plasticizing wheat-milling products containing fractions of bran fiber as filler/reinforcement. Four flours, with different contents of bran fraction, were obtained by sampling along the wheat milling line. The standard alveographic characteristics of reference refined flour allowed the production of film samples, plasticized in the extruder, both with the refined flour F0 and with the milling products F1, F2 and F3 with fiber content of about 15, 25 and 35% wt. The TPWF/bran fiber composites proved to have acceptable mechanical characteristics, which can be improved by the use of suitable quantities of PCL in blend, with citric acid as compatibilizer and with the partial replacement of glycerol with water. Process parameter optimization tests have shown that the lowest plasticization temperature profile (T2) and the highest mixing rate (R120) produced materials with better mechanical properties. In light of the obtained results, we concluded that it is possible to design formulations and manage the process parameters to obtain eco-sustainable and compostable materials from plasticization of raw wheat flour/bran fiber reinforced, at affordable costs, with characteristics designed for different application sectors requiring different mechanical performance.

## Figures and Tables

**Table 1:** Content of plasticizable fraction (PF) and fiber in% wt. of the samples.

Sample	Flour *	Bran **	Plasticizable Fraction (PF)	Unplasticizable Fraction (UF)
F0	100	0	100.0	0.0
F1	80	20	86	14
F2	65	35	75.5	24.5
F3	50	50	65.0	35.0

* FLOUR (PF/UF = 100/0); ** BRAN (PF/UF = 30/70).

**Table 2:** Samples produced: Main constituents and processing parameters.

Sample	Flour	Glycerol (% wt.)	Biopolymer (% wt.)	Citric Acid (% wt.)	T_set_ *	Screw Speed (Rpm)
TPF0	F0	23	0	0	2	30
TPF1	F1	23	0	0	2	30
TPF2	F2	23	0	0	2	30
TPF3	F3	23	0	0	2	30
TPF2_CA	F2	23	0	0.8	2	30
TPF2_20BAT	F2	23	20 PBAT	0	2	30
TPF2_20CL	F2	23	20 PCL	0	2	30
TPF2_CA20BAT	F2	23	20 PBAT	0.8	2	30
TPF2_CA20CL	F2	23	20 PCL	0.8	2	30
F0_CA2	F0	17	0	0.8	2	30
F0_CA3	F0	17	0	0.8	3	30
F0_CA20CL2	F0	17	20 PCL	0.8	2	30
F0_CA20CL3	F0	17	20 PCL	0.8	3	30
F2_CA20CL2	F2	17	20 PCL	0.8	2	30
F2_CA20CL3	F2	17	20 PCL	0.8	3	30
F2_20CL	F2	17	20 PCL	0.8	2	30
F2_20CL120R	F2	17	20 PCL	0.8	2	120
F2_25CL	F2	17	25 PCL	0.8	2	30
F2_25CL120R	F2	17	25 PCL	0.8	2	120
F2_30CL	F2	17	30 PCL	0.8	2	30
F2_30CL120R	F2	17	30 PCL	0.8	2	120
F2_40CL	F2	17	40 PCL	0.8	2	30
F2_40CL120R	F2	17	40 PCL	0.8	2	120
F0_25CL120R	F0	17	25 PCL	0.8	2	120
F1_25CL120R	F1	17	25 PCL	0.8	2	120
F3_25CL120R	F3	17	25 PCL	0.8	2	120

* T_set_ 2 = 130–135–140 °C; 3 = 135–140–145 °C.

**Table 3:** Results of tensile tests made on plasticized flours and their blends with PCL and polybutylene adipate terephthalate (PBAT), by varying the biopolymer amounts, processing temperatures and mixing rate.

Sample	Flour	Glycerol (% wt.)	Biopolymer (% wt.)	Citric Acid (% wt.)	Tset *	Screw Speed (rpm)	Ultimate Tensile Strength (MPa)	Strain at Break (%)
TPF0	F0	23	0	0	2	30	1.23 ± 0.05	54.13 ± 4.39
TPF1	F1	23	0	0	2	30	2.63 ± 0.10	32.23 ± 0.64
TPF2	F2	23	0	0	2	30	2.62 ± 0.40	23.84 ± 2.45
TPF3	F3	23	0	0	2	30	3.83 ± 0.20	19.64 ± 1.60
TPF2_CA	F2	23	0	0.8	2	30	1.61 ± 0,08	34.89 ± 2.44
TPF2_20BAT	F2	23	20 PBAT	0	2	30	2.21 ± 0,17	6.93 ± 0.47
TPF2_20CL	F2	23	20 PCL	0	2	30	1.96 ± 0.06	16.80 ± 1.98
TPF2_CA20BAT	F2	23	20 PBAT	0.8	2	30	1.50 ± 0.05	10.44 ± 0.17
TPF2_CA20CL	F2	23	20 PCL	0.8	2	30	1.66 ± 0.34	60.39 ± 13.00
F0_CA2	F0	17	0	0.8	2	30	1.54 ± 0.02	75.47 ± 11.66
F0_CA3	F0	17	0	0.8	3	30	1.05 ± 0.14	31.08 ± 6.07
F0_CA20CL2	F0	17	20 PCL	0.8	2	30	2.89 ± 0.23	61.80 ± 11,87
F0_CA20CL3	F0	17	20 PCL	0.8	3	30	2.69 ± 0.22	47.04 ± 18.15
F2_CA20CL2	F2	17	20 PCL	0.8	2	30	3,10 ± 0,24	12,42 ± 1,43
F2_CA20CL3	F2	17	20 PCL	0.8	3	30	2,72 ± 0,23	13,83 ± 1,12
F2_20CL	F2	17	20 PCL	0.8	2	30	3.10 ± 0.24	12.42 ± 1.43
F2_20CL120R	F2	17	20 PCL	0.8	2	120	3.17 ± 0.20	14.15 ± 2.46
F2_25CL	F2	17	25 PCL	0.8	2	30	3.05 ± 0.03	16.27 ± 1.47
F2_25CL120R	F2	17	25 PCL	0.8	2	120	3.70 ± 0.16	18.87 ± 2.77
F2_30CL	F2	17	30 PCL	0.8	2	30	3.69 ± 0.43	12.49 ± 1.03
F2_30CL120R	F2	17	30 PCL	0.8	2	120	3.43 ± 0.36	11.73 ± 0.99
F2_40CL	F2	17	40 PCL	0.8	2	30	4,57 ± 0.36	30.75 ± 1.97
F2_40CL120R	F2	17	40 PCL	0.8	2	120	4,63 ± 0.13	52.64 ± 4.02
F0_25CL120R	F0	17	25 PCL	0.8	2	120	2.68 ± 0.11	57.11 ± 6.29
F1_25CL120R	F1	17	25 PCL	0.8	2	120	3.58 ± 0.14	37.39 ± 4.07
F3_25CL120R	F3	17	25 PCL	0.8	2	120	3.91 ± 0.31	10.04 ± 3.92

* T_set_ 2 = 130–135–140 °C; 3 = 135–140–145 °C.

**Table 4:** Germination test results on compost extract taken 40 and 60 days after the incubation.

	40 Days		60 Days	
Samples	G×L	Ig (%)	G×L	Ig (%)
C	37.0		233	
TPF3_50	20.0	54	229	98
TPF1_50	21.0	57	251	108
TPF0_50	9.5	26	183	79
TPF3_75	13.3	36	197	85
TPF1_75	12.5	34	213	91
TPF0_75	1.9	5	171	73

## References

[1] Leblanc N., Saiah R., Beucher E., Gattin R., Castandet M., Saiter J.-M. (2008). Structural investigation and thermal stability of new extruded wheat flour based polymeric materials. Carbohyd. Polym..

[2] Soccio M., Dominici F., Quattrosoldi S., Luzi F., Munari A., Torre L., Puglia D. (2020). PBS-based green copolymer as efficient compatibilizer in Thermoplastic inedible Wheat Flour/Poly (Butylene Succinate) Blends. Biomacromolecules.

[3] Benincasa P., Dominici F., Bocci L., Governatori C., Panfili I., Tosti G., Torre L., Puglia D. (2017). Relationships between wheat flour baking properties and tensile characteristics of derived thermoplastic films. Ind. Crop. Prod..

[4] Puglia D., Dominici F., Kenny J.M., Santulli C., Governatori C., Tosti G., Benincasa P. (2016). Tensile behavior of thermoplastic films from wheat flours as function of raw material baking properties. J. Polym. Environ..

[5] Cano A., Jiménez A., Cháfer M., Gónzalez C., Chiralt A. (2014). Effect of amylose:amylopectin ratio and rice bran addition on starch films properties. Carbohyd. Polym..

[6] Prückler M., Siebenhandl-Ehn S., Apprich S., Höltinger S., Haas C., Schmid E., Kneifel W. (2014). Wheat bran-based biorefinery 1: Composition of wheat bran and strategies of functionalization. LWT-Food Sci. Technol..

[7] ElMekawy A., Diels L., De Wever H., Pant D. (2013). Valorization of Cereal Based Biorefinery Byproducts: Reality and Expectations. Environ. Sci. Technol..

[8] Bressiani J., Oro T., Da Silva P., Montenegro F., Bertolin T., Gutkoski L., Gularte M. (2019). Influence of milling whole wheat grains and particle size on thermo-mechanical properties of flour using Mixolab. Czech. J. Food Sci..

[9] Liu N., Ma S., Li L., Wang X. (2019). Study on the effect of wheat bran dietary fiber on the rheological properties of dough. Grain Oil Sci. Technol..

[10] De Bondt Y., Liberloo I., Roye C., Goos P., Courtin C.M. (2020). The impact of wheat (Triticum aestivum L.) bran on wheat starch gelatinization: A differential scanning calorimetry study. Carbohyd. Polym..

[11] Dobircau L., Sreekumar P.A., Saiah R., Leblanc N., Terrié C., Gattin R., Saiter J.M. (2009). Wheat flour thermoplastic matrix reinforced by waste cotton fibre: Agro-green-composites. Compos. Part A-Appl. S..

[12] Pérez-Pacheco E., Canto-Pinto J.C., Moo-Huchin V.M., Estrada-Mota I.A., Estrada-León R.J., Chel-Guerrero L. (2016). Thermoplastic Starch (TPS)-Cellulosic Fibers Composites: Mechanical Properties and Water Vapor Barrier: A Review. Compos. Renew. Sustain. Mater..

[13] Hanis-Syazwani M., Bolarinwa I.F., Lasekan O., Muhammad K. (2018). Influence of starter culture on the physicochemical properties of rice bran sourdough and physical quality of sourdough bread. Food Res..

[14] Roozendaal H., Madian A., Frazier R.A. (2012). Thermogravimetric analysis of water release from wheat flour and wheat bran suspensions. J. Food Eng..

[15] El-Sayed S. (2019). Thermal decomposition, kinetics and combustion parameters determination for two different sizes of rice husk using TGA. Eng. Agric. Environ. Food.

[16] Yu A.-N., Li Y., Yang Y., Yu K. (2018). The browning kinetics of the non-enzymatic browning reaction in L-ascorbic acid/basic amino acid systems. Food Sci. Technol..

[17] Edoardo Capuano E., Ferrigno A., Acampa I., Ait-Ameur L., Fogliano V. (2008). Characterization of the Maillard reaction in bread crisps. Eur. Food Res. Technol..

[18] Rosell C.M. (2011). The Science of Doughs and Bread Quality. Flour and Breads and Their Fortification in Health and Disease Prevention.

[19] Majewsky L., Cunha A.G. (2018). Evaluation of Suitability of Wheat Bran as a Natural Filler in Polymer Processing. Bioresources.

[20] Follain N., Joly C., Dole P., Roge B., Mathlouthi (2006). M. Quaternary starch based blends: Influence of a fourth component addition to the starch/water/glycerol system. Carbohydr. Polym..

[21] Dominici F., Gigli M., Armentano I., Genovese L., Luzi F., Torre L., Munari A., Lotti N. (2020). Improving the flexibility and compostability of starch/poly(butylene cyclohexanedicarboxylate)-based blends. Carbohydr. Polym..

[22] Carvalho A.J.F., Zambon M.D., da Silva Curvelo A.A., Gandini A. (2005). Thermoplastic starch modification during melt processing: Hydrolysis catalyzed by carboxylic acids. Carbohydr. Polym..

[23] Genovese L., Dominici F., Gigli M., Armentano I., Lotti N., Torre L., Munari A. (2018). Processing, thermo-mechanical characterization and gas permeability of thermoplastic starch/poly(butylene trans-1,4-cyclohexanedicarboxylate) blends. Polym. Degr. Stab..

[24] Olivato J.B., Grossmann M.V.E., Yamashita F., Eiras D., Pessan L.A. (2012). Citric acid and maleic anhydride as compatibilizers in starch/poly(butylene adipate-co-terephthalate) blends by one-step reactive extrusion. Carbohydr. Polym..

[25] Jiugao Y., Ning W., Xiaofei M. (2005). The Effects of Citric Acid on the Properties of Thermoplastic Starch Plasticized by Glycerol. Starch/Stärke.

[26] Lagrain B., Thewissen B.G., Brijs K., Delcour J.A. (2008). Mechanism of gliadin–glutenin cross-linking during hydrothermal treatment. Food Chem..

[27] Basiak E., Lenart A., Debeaufort F. (2018). How Glycerol and Water Contents Affect the Structural and Functional Properties of Starch-Based Edible Films. Polymers.

[28] Khamthong P., Lumdubwong N. (2012). Effects of heat-moisture treatment on normal and waxy rice flours and production of thermoplastic flour materials. Carbohydr. Polym..

[29] Jbilou F., Ayadi F., Galland S., Joly C., Dole P., Belard L., Degraeve P. (2012). Effect of Shear Stress Extrusion Intensity on Plasticized Corn Flour Structure: Proteins Role and Distribution. J. Appl. Polym. Sci..

[30] Sasimowski E., Majewski L., Grochowicz M. (2019). Influence of the Design Solutions of Extruder Screw Mixing Tip on Selected Properties of Wheat Bran-Polyethylene Biocomposite. Polymers.

[31] Carmona V.B., Corrêa A.C., Marconcini J.M., Mattoso L.H.C. (2014). Properties of a Biodegradable Ternary Blend of Thermoplastic Starch (TPS), Poly(ε-Caprolactone) (PCL) and Poly(Lactic Acid) (PLA). J. Polym. Environ..

[32] Sin L.T., Rahman W.A.W.A., Rahmat A.R., Mokhtar M. (2011). Determination of thermal stability and activation energy of polyvinyl alcohol-cassava starch blends. Carbohydr. Polym..

